# Hepatocyte ballooning and steatosis in early and late gestation without liver malfunction: Effects of low protein/high carbohydrate diet

**DOI:** 10.1371/journal.pone.0294062

**Published:** 2024-01-02

**Authors:** Mónica Navarro-Meza, Mauricio Díaz-Muñoz, José Alfonso Cruz-Ramos, Jonathan Rafael Trinidad Gallardo, María Conchita Rodríguez Oseguera, Paola C. Bello-Medina, Ericka Alejandra De Los Ríos-Arellano

**Affiliations:** 1 Departamento de Promoción, Preservación y Desarrollo de la Salud, División Ciencias de la Salud, Laboratorio C de Memoria y Neuronutrición. Centro Universitario del Sur, Universidad de Guadalajara, Ciudad Guzmán, Jalisco, México; 2 Departamento de Neurobiología Celular y Molecular, Instituto de Neurobiología, Universidad Nacional Autónoma de México, Querétaro, México; 3 Departamento de Clínicas Médicas, Universidad de Guadalajara, Guadalajara, Jalisco, México; 4 Coordinación de Investigación, Subdirección de Desarrollo institucional, Instituto Jalisciense de Cancerología, Guadalajara, Jalisco, México; 5 Facultad de Ciencias e Instituto de Educación a distancia IDEAD, Universidad del Tolima, Ibagué, Tolima, Colombia; St Jude Children’s Research Hospital, UNITED STATES

## Abstract

Pregnancy is a challenging metabolic and physiological condition. The aim of this study was to include a second demanding situation as a low protein/high carbohydrate diet (LPHCD) to characterize the histological and functional responses of the maternal liver. It is unknown how the maternal liver responds during early and late pregnancy to LPHCD intake. We explored early pregnancy (3 and 8 gestational age, G) and late pregnancy (15 and 20 G). The results indicated that pregnant rats under control diet showed an evident presence of ballooned hepatocytes, lipid vesicles and edema at late pregnancy (15G); in contrast, pregnant rats under LPHCD showed similar pattern of histological modification but at early pregnancy (3G). Unexpectedly, the serum biomarkers didn’t display functional alterations in either group, despite of the evident histological changes no liver malfunction was detected. We conclude that pregnant rats fed with control diet and experimental LPHCD, are subjected to metabolic and physiological conditions that impact the histopathological condition of the maternal liver. Control diet promoted the histological modifications during late pregnancy whereas LPCHCD advanced the onset of these changes. Further experiments are needed to explore the biochemical mechanisms that underlie these histological modifications. Our results are also an example of the resilience associated with the pregnancy: since no functional hepatic alterations accompanied the histopathological changes, another conclusion is that no evident pathological condition was detected in this nutritional protocol.

## 1. Introduction

During pregnancy, the liver regulates intermediate metabolic pathways, as well as the anabolic reactions that synthesize glucose, lipids, and ketones needed to meet energy requirements [1, 2]. This process is a rheostatic mechanism in which the maternal body adapts to environment changes [3]. A progressive accumulation of fatty acid deposits and an increase of adipose tissue related to lipogenesis occurs in early pregnancy; this event is associated with the anabolic process experienced during the first half of pregnancy [4]. Studies have reported an accumulation of liver cholesterol and triacylglycerides (TAGs) during the gestational period, as a mechanism involved in the regulation of proper fetal development in cases of maternal malnourished conditions [2, 5]. Additionally, in response to overfeeding with high-fat diets, and obesity there is an increase of FFA acid uptake by the liver, as well as an increased lipogenesis and a decreased FA oxidation, leading to ectopic triglyceride deposition in the liver [6]. Notwithstanding, the understanding of liver lipid metabolism during pregnancy remains incomplete. In the last 10 years, the prevalence of hepatic steatosis in pregnancy has tripled worldwide. Furthermore, it is estimated that 10% of women of reproductive age in Western and Asian countries suffer from this condition [7]. The likelihood of developing this disorder is higher in Hispanic women, especially in those with higher parity [8]. When assessing steatosis, hepatocyte ballooning has been one of the means used to identify potential progression from simple steatosis to steatohepatitis. It is defined as a cellular enlargement of 1.5 to 2 times the standard hepatocyte diameter, with loss of the usual and peculiar polygonal shape of cells combined with pale cytoplasm [9, 10]. Nonetheless, the relationship between steatosis development and hepatocyte ballooning formation during early and late pregnancy and LPHCD remains unclear. Previous studies by our research group indicated that LPHCD, consumed by primiparous mothers during pregnancy, promotes the development of fatty vesicles in the liver, suggesting that this condition could be due to metabolic adaptations and nitrogen management reflected in reduced serum urea levels and impaired amino acid profiles in the liver [11]. However, it’s not clear the maternal liver response to LPHCD during pregnancy, and if these events could result in liver damage. This study analyzed whether LPHCD intake during gestation promotes liver histology and changes in serum markers in the early and late stages. We found modifications in liver histology due to pregnancy, which also occur when the experimental diet is consumed in different settings. These modifications do not affect the proper functioning of the maternal liver.

## 2. Materials and methods

### 2.1 Ethical approval

The procedures were carried out in accordance with the Official Mexican Standard: NOM-062-ZOO-1999. This research was reviewed and authorized by the Ethics Committee of the Neurobiology Institute, at the Universidad Nacional Autónoma de México (UNAM) (approval ID 081. A).

### 2.2 Experimental groups

The female rats were individually held in cages (21×23.5×38 cm) under controlled 12 h dark/light conditions with lights on at 07:00 h. A progressive study was performed in mothers during gestation (G) days (3, 8, 15, and 20). Early pregnancy was considered the 3rd to 8th day of gestation, and late pregnancy from the 15th to 20th day. Fifty-eight female Wistar rats weighing 250–300 g, were randomly divided into 4 groups:

1. Non-pregnant rats fed with control diet (CNP): n = 5 (for a period of 7 days).
2. Non-pregnant rats fed with an experimental (LPNP) isocaloric and protein restricted/high carbohydrate diet (LPHCD) containing 6% protein n = 5 (for a period of 7 days).
3. Pregnant rats fed with control diet (CP): n = 24, 6 rats in each day. Early pregnancy (3G and 8G); late pregnancy (15G and 20G).
4. Pregnant rats fed with an experimental (LPP) isocaloric and protein restricted/high carbohydrate diet containing 6% protein: n = 24, 6 rats in each day. Early pregnancy (3G and 8G); late pregnancy (15G and 20G).

#### 2.2.1. Diet and body weight recording

A control diet (AIN-93G Growth Purified Diet, 57W5, *Test Diet*) (18.3% protein, 63.2% carbohydrate, 7.1% fat at 3.90 kcal/g) and an experimental diet (AIN-93G w/ 6.0% Total Protein, 5T0Q, *Test Diet*) (6.0% protein, 77.3% carbohydrate, 7.1% fat at 3.97 kcal/g) were used. Nutritional composition of diets is illustrated in Table 1. All groups had ad libitum access to diets and water intake. Even though both diets are isocaloric, there are evident differences between them: the experimental diet has 67.2% less protein and 18.2% more carbohydrate. Before and during pregnancy, food and water intake were quantified, and body weight was registered using a precision scale (A&D Weighing series GF-3000 scale).

**Table 1:** Nutritional composition of control and experimental diets.

Nutrient	Normal protein diet (%)	Low protein diet/high carbohydrate diet (%)
**Protein**	**18.3**	**6.0**
Casein-Vitamin tested	20	5.9
**Carbohydrate**	**63.2**	**77.3**
Corn Starch	39.74	53.81
Maltodextrin	13.20	13.20
Sucrose	10	10
**Fat**	**7.1**	**7.1**
Total Saturated Fatty A	1.05	1.05
Total Monounsaturated fatty acids	1.54	1.54
Polyunsaturated fatty acids	3.78	3.78
**Minerals**	**3.500**	**3.500**
Calcium	0.51	0.51
Phosphorus	0.32	0.21
Potassium	0.36	0.36
Magnesium	0.05	0.05
Sodium	0.13	0.14
Chloride	0.22	0.22
Fluorine, ppm	1.0	1.0
Iron, ppm	40	39
Zinc, ppm	35	30
Manganese, ppm	11	11
Copper, ppm	6.0	6.0
Cobalt, ppm	0.0	0.0
Iodine, ppm	0.21	0.21
Chromium, ppm	1.0	1.0
Molybdenum, ppm	0.14	0.14
Selenium, ppm	0.24	0.19
**Vitamins**	**1.0000**	**1.0000**
Vitamin A, IU/g	4.0	4.0
Vitamin D-3 (added), IU/g	1.0	1.0
Vitamin E, IU/kg	81.6	81.6
Vitamin K, ppm	0.75	0.75
Thiamin Hydrochloride, ppm	4.8	6.0
Riboflavin, ppm	6.7	6.2
Niacin, ppm	30	30
Pantothenic Acid, ppm	16	15
Folic Acid, ppm	2.1	2.0
Pyridoxine, ppm	5.8	5.8
Biotin, ppm	0.2	0.2
Vitamin B-12, mcg/kg	28	26
Choline Chloride, ppm	1,250	1, 250
Ascorbic Acid, ppm	0.0	0.0

#### 2.2.2. Liver tissue and serum sampling

Before animal’s diseccion the CO_2_ anesthesia was used to suppress the experimental animals suffering. All the sacrifice procedure was approved by the Bioethical Committee of the Neurobiology Institute and followed the Mexican norm for care and management of experimental animals NOM 062-ZOO-1999 after quickly the rats were decapitated with a guillotine to obtain blood samples; tissues were dissection, weighed for biochemical and histological processing. Blood was collected in Eppendorf tubes and centrifuged at 400×g for 10 min to obtain the serum, which was stored in aliquots at −20 °C until analysis. Dissected liver tissues were obtained and processed for histological analysis. Also, adipose tissue was collected.

### 2.3 Histological techniques

Liver tissue was dissected and weighted, then, a representative liver sample was fixed in 10% neutral buffered formalin, dehydrated in alcohol solutions and later embedding in paraffin, sectioned at 3 μm of thickness in a rotation microtome (Leica RM2135, Leica Microsystems, Nussloch, Germany), and finally stained with hematoxylin and eosin method. A trained pathologist determined the hepatic morphological and histological characteristics, such as the number of fat micro and macro vesicles, necrosis and inflammation [12]. The presence of ballooning degeneration was evaluated through microscopic observation with the staining of hematoxylin-eosin, it was determined by the increase in hepatocyte diameter two to three times of its normal size, which consists of the swelling of the cytoplasm due to increased water or lipid content, which is visualized by formation of scattered microvacuoles, which tend to merge and accompany themselves with an eosinophilic network that represents intermediate filaments that show a disruption related to the pathological process.

### 2.4 Biochemical determination in serum

Alanine aminotransferase (ALT/GPT) and aspartate aminotransferase (AST/GOT) assays were carried out on a Spinreact Spin 120 automated equipment for colorimetric assays. In order to measure the insulin levels, enzyme- linked immunosorbent assay (ELISA) kits for insulin serum [American Laboratory Products Company (ALPCO) for the North American life science markets (Cat. 80-INSHU-E01.1)] were used. To determine serum urea and creatinine concentrations, colorimetric methods were used with commercial kits (Spinreact, Santa Coloma, Spain). The values of glucose, the lipid profile, cholesterol levels, and triglycerides were also determined using (Spinreact) commercial kits for colorimetric assays.

### 2.5 Statistical analysis

Statistical analyses were performed with the SPSS Statistics software (IBM, Armonk, NY, USA), considering a value of *P<0*.*05* as statistically significant. The Kolmogorov-Smirnov and the Shapiro Wilk tests were used to calculate the normality of the experimental variables. To compare non-pregnant rats fed with the control and low protein diets, repeated measures two-way ANOVA were used to compare gestational time (factor 1) and diet (factor 2) variations of the pregnant rats in the control group and the ones in the experimental group. Bonferroni post hoc test was used when appropriate; *P < 0*.*05* was considered statistically significant.

## 3. Results

### 3.1 Morphometric study

**Fig 1:**
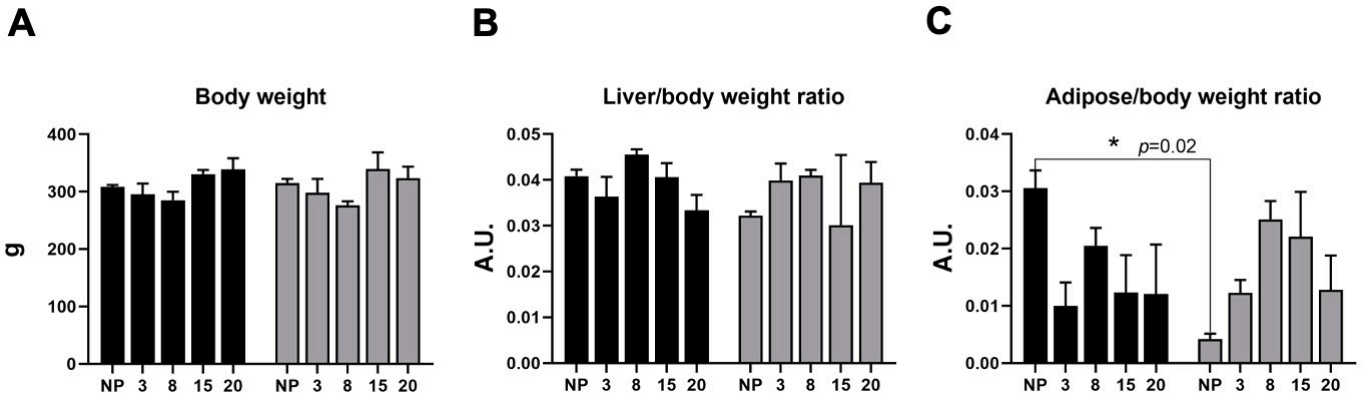
Morphometric liver characteristics in non-pregnant (n = 10) and pregnant rats (n = 48) at different stages of pregnancy. Gray bars correspond to the low protein/high carbohydrate diet (LPHCD) group; black bars correspond to the control group (CNP, CP). Shows proportion in arbitrary units (A.U.). A) Body weight, B) Liver/Body weight ratio, C) Adipose/body weight ratio. Data are shown as mean±standard error; and *P<0.*05*. **P<0.*01*, ***P<0.*0001*.

There were no differences between the body weight before and during pregnancy on female rats (Fig 1A).

There was no difference observed in the liver/body weight ratio between early and late pregnancy (Fig 1B). Adipose/body weight ratio (Fig 1C) was increased in the NP control group compared to the experimental group by 86% (P = 0.02).

### 3.2 Histology (H&E)

**Fig 2:**
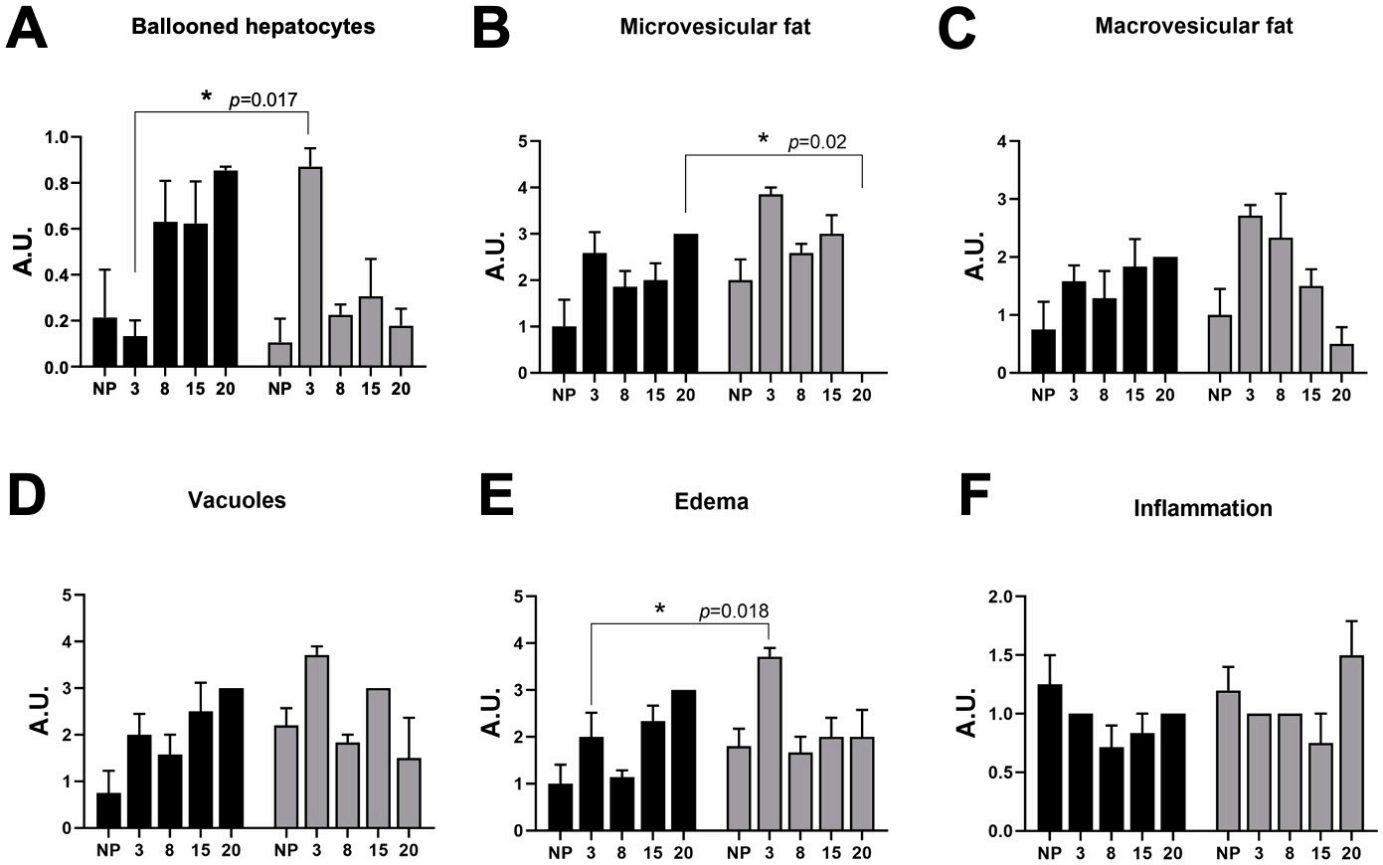
Liver histology by hematoxylin and eosin (H&E) in non-pregnant (n = 10) and pregnant rats (n = 48) at different stages of pregnancy. Pathological evaluation of the liver. Gray bars correspond to the low protein/high carbohydrate diet (LPHCD) group; black bars correspond to the control group (CNP, CP). Shows proportion in arbitrary units (A.U.). A) Ballooned hepatocytes item, B) Fat microvesicles item, C) Fat macrovesicles item, D) Vacuoles item, E) Edema item, F) Inflammation item. Data are shown as mean±standard error; and *P<0.*05*. **P<0.*01*, ***P<0.*0001*.

**Fig 3:**
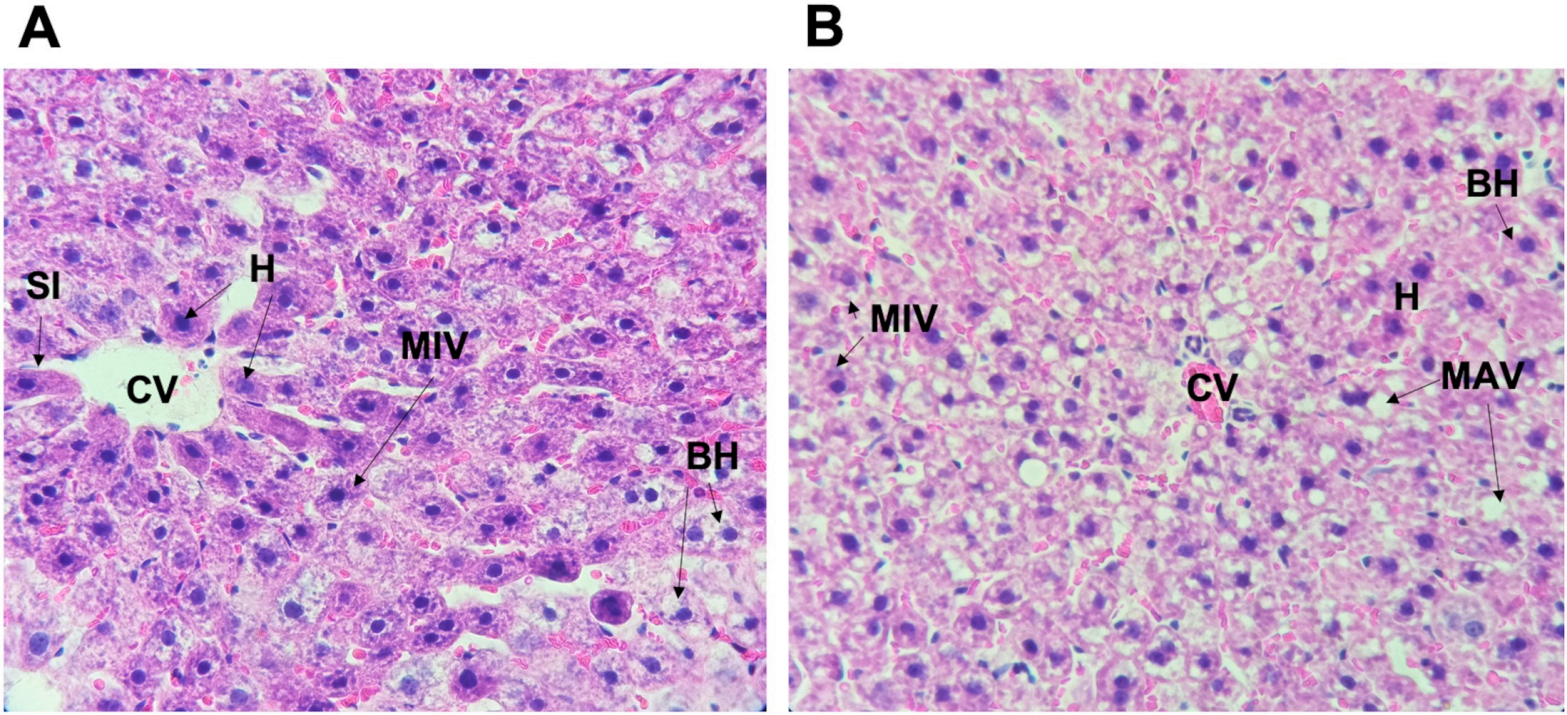
Liver histology by hematoxylin and eosin (H&E) in non-pregnant rats. **A. Liver biopsy of control non pregnant rat (CNP).** Accumulation of fat micro vesicles (MIV) was **ts.**observed in the cytoplasm. No damage in central vein (CV) and sinusoids (SI), normal hepatocytes (H), and trabeculae were observed. Ballooned hepatocytes (BH) were detected. **B. Liver biopsy of non-pregnant rats fed with experimental diet (LPHCD)**. Diffuse accumulation of fat micro vesicles (MIV) and macrovesicles (MAV) were observed in the cytoplasm. Ballooning degeneration (BH) was also noticed.

Nulliparous liver biopsies demonstrated accumulation of fat microvesicles both in the control group (CNP) and experimental group (LPNP) (Fig 2B) without damage in the central vein (Fig 3). The count of ballooned hepatocytes demonstrated a higher quantity on the nulliparous control group than the LPNP group (Fig 2A) even though it was not statistically significant.

We observed an increase of 46% in edema in the experimental group (P = 0.018) contrasted with the control group (Fig 2F) in the early pregnancy stage (3G). In the control group, the presence of edema stood out at week 20. Regarding the vacuoles (Fig 2D), in the experimental group the quantity of these stood out in week 3, while in the control group the greatest presence of vacuoles was observed at week 20. There was no statistical difference.

Ballooned hepatocytes were counted (Fig 3A) then their intergroup and intragroup correlations were analyzed using Bonferroni’s multiple comparisons test. We found a significant increase of ballooned hepatocytes in early pregnancy (3G) in the experimental group (LPHCD) in contrast with the control group in the same stage of pregnancy *(P = 0*.*001)*.

(Fig 2A). Interestingly, this behavior was invested at the end of pregnancy, where the control group showed four labels increase in the balloon hepatocyte count compared to LPHCD group. When comparing the NPLP versus 20G control group, an increase of eight labels was found (p = 0.0327). Then, comparing NPLP vs. 3G LPHCD, an increase of eight labels (A.U) of ballooned hepatocytes was found *(P = 0*.*0115)*. When comparing 3G LPHCD vs. 20G LPHCD (early pregnancy versus late pregnancy in experimental group), a significant decrease of four labels (AU) of ballooned hepatocytes was observed by the end of pregnancy (p = 0.032), this behavior was also invested in the control group. Comparing the 3G control group versus 20G control group (early pregnancy versus late pregnancy in control group), an increase of eight labels (AU) was found (P = 0.047).

**Fig 4:**
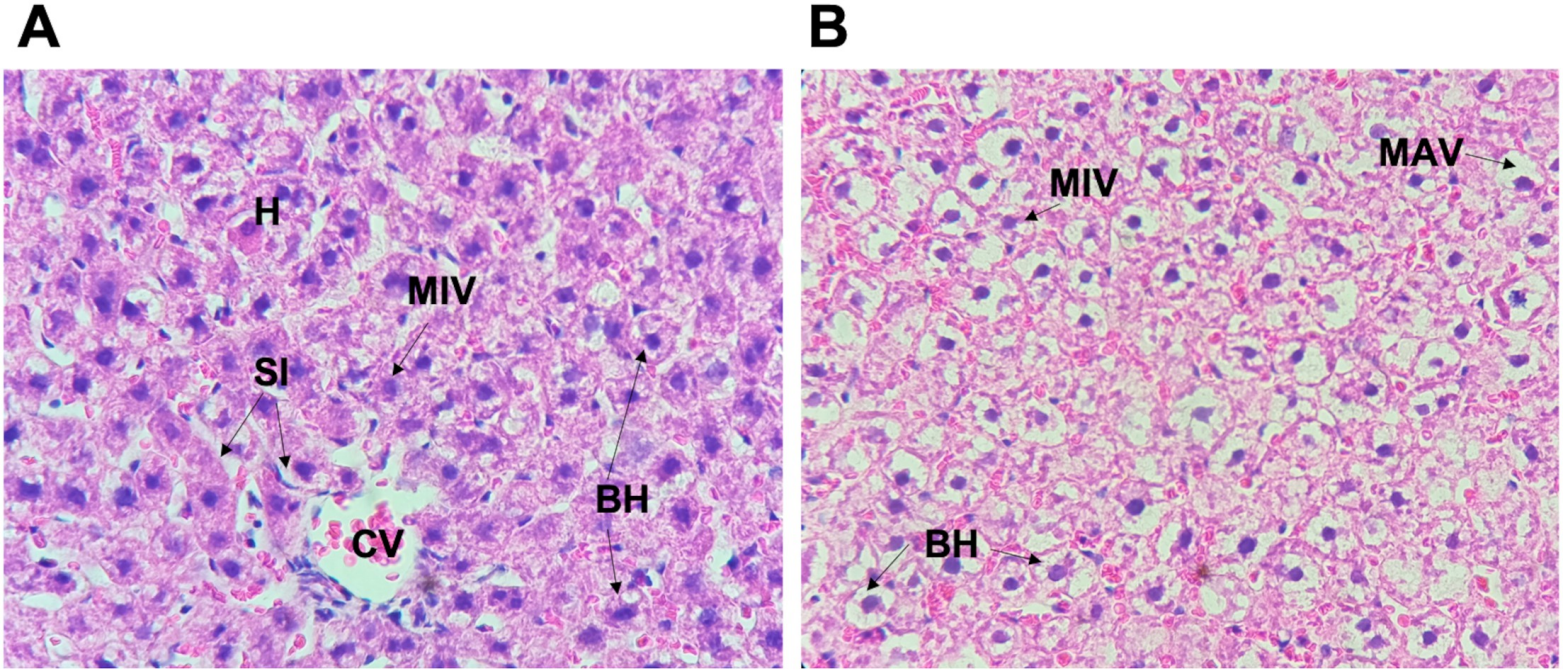
Liver histology in early pregnant rats (hematoxylin-eosin). **A. Liver biopsy of pregnant rat on the control diet group, at the third day of gestation (3G).** Accumulation of fat microvesicles (MIV) were observed in the cytoplasm. No damage in central vein (CV) and sinusoids (SI), normal hepatocytes (H), and trabeculae were reported. Ballooned hepatocytes (BH) were also observed. **B. Liver biopsy of pregnant rat on the low protein diet group, at the third day of gestation (3G).** Diffuse accumulation of fat microvesicles (MIV) and macrovesicles (MAV) were observed in the cytoplasm. Several hepatocytes showing ballooning degeneration (BH) were also observed.

**Fig 5:**
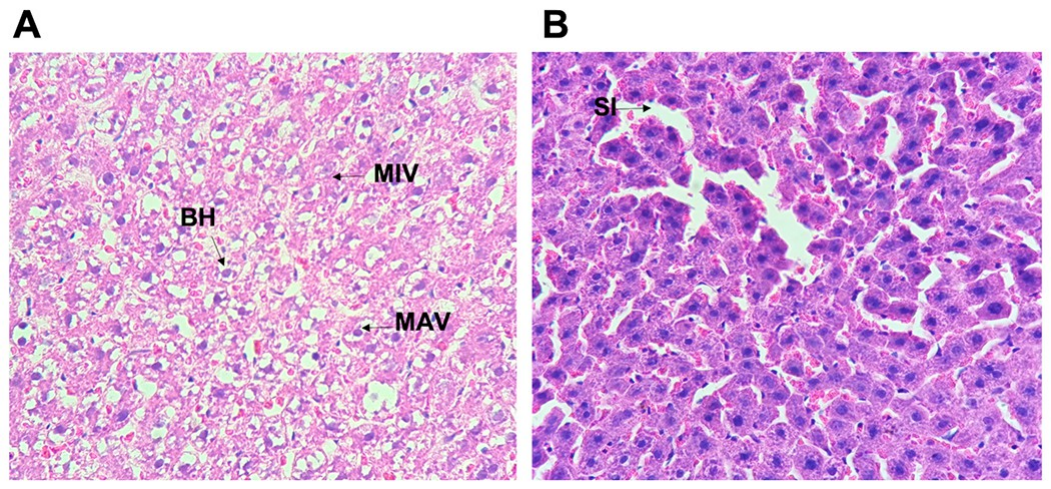
Liver histology in late pregnant rats (hematoxylin-eosin). **A. Liver biopsy of pregnant rat on the control diet group, at twentieth day of gestation (20G).** Diffuse accumulation of fat microvesicles (MIV) and macrovesicles (MAV) were largely observed in the cytoplasm (steatosis). Ballooned hepatocytes (BH) were also observed. **B. Liver biopsy of pregnant rat on the low protein diet group, at twentieth day of gestation (20G).** No accumulation of fat microvesicles were observed, normal hepatocytes were observed, nevertheless, it is observed notable sinusoidal (SI) dilatation.

In the experimental group (LPHCD), we observed an increase in fat micro vesicles, primarily in early pregnancy (3G) (Figs 2B and 4), however, on day 20G corresponding to late pregnancy, no micro vesicles were observed (Figs 2B and 5). Regarding the control group, this tendency to increase was also identified, likewise in this group the presence of micro vesicles was present even on day 20 (Fig 5A), so this difference between both groups was statistically significant (P = 0.02) at this stage of late pregnancy. In relation to macrovesicles, a similar behavior was noticed, the highest number of macrovesicles was detected in the early pregnancy stage (3G) with a decreasing trend until late pregnancy (Fig 2C). In the control group this behavior was not observed, being the late pregnancy stage (20G) the one that presented the highest number of macrovesicles (Figs 2C and 5); although, neither difference was statistically significant.

The necrosis was also evaluated. Regarding the experimental group, the same behavior as the macrovesicles was observed, with a peak in the early pregnancy stage (3G) and a tendency to decrease as the pregnancy progressed. In the control group, the early and late stages of pregnancy were those with the greatest presence of necrosis; however, neither difference was statistically significant. Regarding inflammation, it was observed that in the control group the maximum corresponded to nulliparous females, while in the experimental group the maximum was observed 20G, however, no difference was significant.

### 3.3 Biomarkers in serum

**Fig 6:**
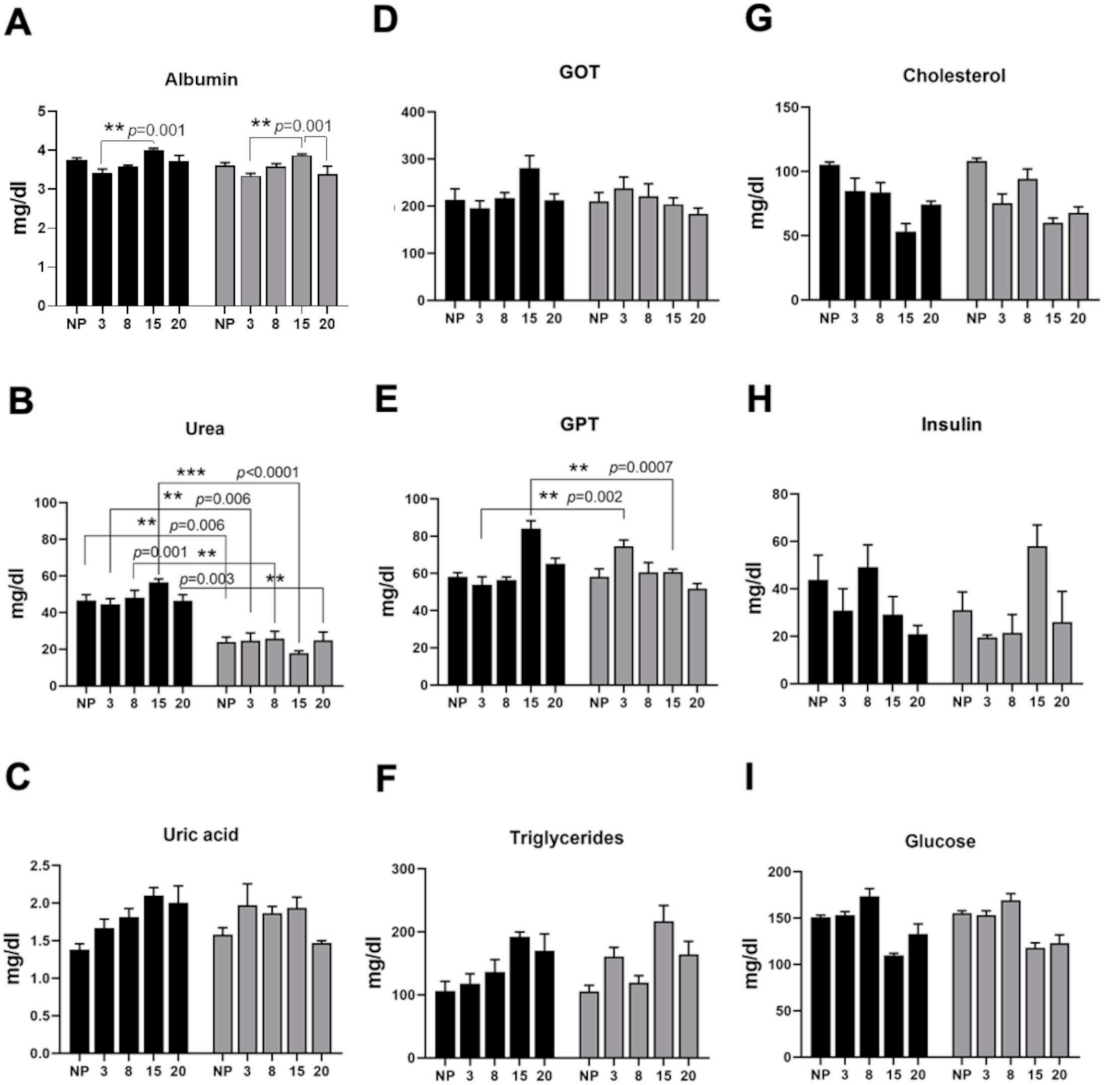
Biomarkers in serum of non-pregnant (NP) (n = 10) and pregnant rats (n = 48) at different stages of pregnancy. Gray bars correspond to the low protein/high carbohydrate diet (LPHCD) group; black bars correspond to the control group (CNP, CP). Albumin (A), Urea (B), Uric acid (C), Glutamic oxaloacetic transaminase (GOT) (D), Glutamic pyruvic transaminase (GPT) (E), Triacylglycerides (F), Cholesterol (G), Insulin (H), Glucose (I). Data are shown as *mean±standard error*; and *P<0.*05*. **P<0.*01*, ***P<0.*0001*.

The temporal profiles of all the serum parameters studied in this project are included in Fig 6. Not all the parameters measured showed significant changes, due to either the pregnancy process or consumption of the LPHCD.

The cholesterol levels (Fig 6G) did not present statistically significant changes; nonetheless, a certain tendency to decrease cholesterol was observed on the 15G day of pregnancy in both groups. Serum levels of LDL and cholesterol were maintained with slight variations throughout the gestation, though the amount of LDL on day 3 of gestation was statistically significant *(P = 0*.*01)* both in the experimental and in the control group, increased in this last group.

No statistically significant changes in serum glucose levels were found (Fig 6I). There is a certain tendency to decrease glucose in late pregnancy (15G and 20G) in both groups. With respect to transaminases, the only parameter with a relevant difference was GPT (Fig 6E). Captivatingly, during the initial stage of pregnancy (3G), a noteworthy elevation *(P = 0*.*002)* in the level of GPT was detected in the experimental group. Conversely, by week 20, a significant increase *(P = 0*.*0007)* in GPT concentration was identified in the control group in comparison to the experimental group. During the latter stages of pregnancy (15G and 20G) there was an observed tendency towards increased levels of triglycerides in both groups (Fig 6F), yet no statistically significant differences were found. Significant intergroup differences were described in urea levels in each of the stages of pregnancy (Fig 6B), in addition to nulliparous females. At each stage, a notable decrease in urea concentration was observed in the experimental groups compared to the control groups (Fig 6B); in spite of that, these results were expected in the experimental group due to the low protein content in the diet and the urea metabolism. No statistically significant changes in serum insulin levels were detected (Fig 6H); notwithstanding, both groups exhibit behavior indicating an insulin peak during the transition from early to late pregnancy. The levels of albumin (Fig 6A), creatinine, uric acid (Fig 6C) and GOT (Fig 6D) did not show significant differences between groups or throughout the pregnancy.

## 4. Discussion

Pregnancy is a rheostatic mechanism where metabolic changes occur in maternal environment [3, 5]. Little is known about LPHCD intake and histological changes in maternal liver. In this study we observed liver histological changes during early and late pregnancy related to LPHCD in maternal liver. Our data showed the pregnancy itself is enough to elicit histological adaptations in the maternal liver. It’s known during early pregnancy the maternal liver related a progressive infiltration of fatty acids and increased lipid metabolism [4, 5]. During gestation, lipid homeostasis may be altered because of the association of insulin resistance proper of pregnancy and excessive lipid accumulation in non-adipose tissues, particularly muscle and liver [13].

### 4.1 Morphometric study

Studies indicate protein restriction during pregnancy does not alter mothers body weight and does not affect birth [14]; our results are consistent, we founded that LPHCD intake did not show changes in body weight in females mothers compared with control groups and different times of pregnancy. Liver weight did not show changes either, we propose that this diet didn’t compromise liver morphology. However, in other studies reported that the LPD intake during pregnancy promotes liver enlargement caused by an increase of hepatic triglycerides [15, 16]. This event could be related to quantity and content of nutrients and energy proportion of the diet.

### 4.2 Histological changes

Previous studies showed that LPHCD intake during pregnancy promotes the development of fatty vesicles in liver of primiparous mothers [11]. Our study showed the control diet used in maternal liver during pregnancy presented an important increase of ballooned hepatocytes, edema, micro and macro lipid vesicles mainly in late pregnancy. Similar histological adaptations related to LPHCD intake were observed at earlier pregnancy stages.

The accumulation of fat is recognized as a critical factor in fetal growth during the second half of gestation [17], since FA are essential biomolecules to generate energy substrates [18]. The excessive release of FAs as a product of enhanced lipolysis is uptake by other organs, mainly liver and skeletal muscle, which are not equipped to safely store this excess of fat, this can induce a progression of metabolic changes. Our results suggest a possible enhanced fat mobilization and utilization of fat depots by the experimental group, because contrary to the control group, fat depots were diminished upon late pregnancy. Supporting this, other studies have demonstrated that LPD intake during pregnancy decreases de novo lipogenesis and increases FA oxidation [19].

The macro vesicular accumulation is characterized by single large lipid droplet as well as the displacement of the nucleus towards the periphery. On the other hand, micro vesicular accumulation is characterized by the presence of small lipid droplets without causing nuclear displacement [20]. It has been suggested that micro vesicular accumulation could be affected by mitochondrial dysfunction and subsequently defects in beta-oxidation. Several studies propose that macro vesicular accumulation is associated with increased proliferation of hepatocytes, suggesting that it could be considered a "benign" type of steatosis, in contrast to macro vesicular steatosis, and its association with decreased hepatocyte proliferation, however the foundations of this notion are still not entirely clear [20]. In our study we observed an accumulation of micro vesicles and macro vesicles, with a clear increase in early pregnancy (3G) in both control and experimental groups, but it was notably higher in LPHCD group. The accumulation of fat deposits has been reported by other studies [2–5] suggesting it is due to lipogenesis and glycerol neogenesis [3, 4] depending on anabolic stages. However, the dynamics of fat accumulation in liver during early and late pregnancy under physiological conditions remains unclear.

Hepatocellular ballooning is considered a form of damage in the liver. Fluid retention, the amount and conformation of intracellular organelles and cytoplasmic components are believed to have an impact on cell size and structure, leading to the characteristic ballooned shape [21, 22]; however, its development during pregnancy is poorly understood. The widely accepted “two-hit hypothesis” considered steatosis to be the first hit, which increases the sensitivity of the liver to second hits, leading to hepatocyte injury and eventually inflammation and fibrosis [23, 24]. According to new evidence, we suggest that these changes could result from an interaction between environmental influences, epigenetic and genetic variations, mitochondrial dysfunctions, oxidative stress and lipid metabolism deregulation [23, 24]. In our study we observed the relation between increased of micro and macrovesicles with ballooning. Curiously, we noted an inverse relation regarding LPHCD group, where we found a clear decrease in ballooning degeneration upon late pregnancy and a downturn of fat accumulation. We believe this decrease of fat accumulation could be preventing the progression of ballooning degeneration in experimental group upon late pregnancy.

### 4.3 Biomarkers in serum

Serum biomarkers did not showed changes in both groups, except for urea and GPT (Fig 6B and 6E). The former was expected due the low protein content in diet and the urea metabolism, and even though GPT was elevated, it was not enough to suggest functional liver compromise. We did not find significant changes in other hepatic serum biomarkers. These results are consistent with other studies with protein restriction, where biochemical and hematological parameters were not altered, and seemingly not to have any damage effect on pregnant rats [25].

### 4.4 Molecular mechanisms of liver diseases associated with gestational diabetes

Nevertheless, we did not detect any liver functional dysregulation, it is pertinent to consider cellular and metabolic mechanisms that underlie gestational diabetes and liver steatosis:

Gestational diabetes is mainly associated with high hepatic liver production by gluconeogenesis. The liver production of glucose in is the result of an equilibrium between the glucose uptake and the gluconeogenic activity, being regulated by diverse signaling pathways [26, 27]. It has been reported that adiponectin deficiency leads to liver steatosis during pregnancy, condition that is associated with glucose intolerance and altered gluconeogenesis [26]. In contrast, high levels of adiponecting during late pregnancy attenates lipid deposits and restored glucose homeostasis [28].

## 5 Conclusion

Pregnancy is a rheostatic state characterized by histological hepatic modifications at late stages, specifically in the enhanced number of ballooned hepatocytes, and the presence of lipid vesicles, when the mother’s intake of low protein, high carbohydrate diet these adaptations appeared in early state and in spite of the evident histological changes, no malfunction of liver activity was detected.

## Supporting information

S1 Graphical abstract (TIF)

## Abbreviations

AFLP: Acute fatty liver of pregnancy
ALT: alanine transaminase
ATP: Adenosine triphosphate
FA: Fatty acids
FFA: Free fatty acids
GOT: glutamic-oxaloacetic transaminase
GPT: glutamate pyruvate transaminase
H&E: Hematoxilin and eosin staining
LPD: Low Protein Diet
LPHCD: low protein/high carbohydrate diet
NAFLD: nonalcoholic fatty liver disease
NASH: nonalcoholic steatohepatitis
TAGs: triacylglycerides
