# Supplementary figures and images for: Hepatocyte ballooning and steatosis in early and late gestation without liver malfunction: Effects of low protein/high carbohydrate diet
Source: PLoS One. 2024 Jan 2;19(1):e0294062. doi: 10.1371/journal.pone.0294062 (PMC10760903; doi:10.1371/journal.pone.0294062)

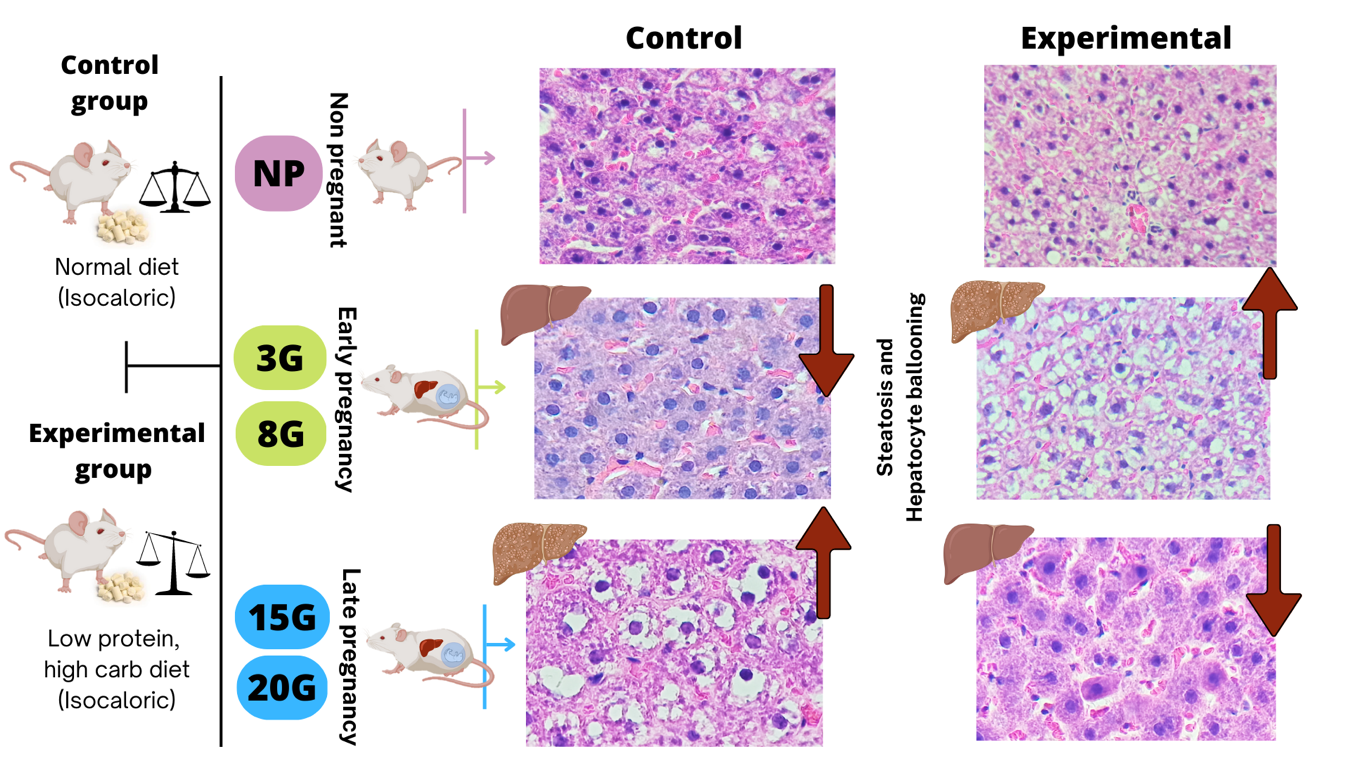

Supplement: S1 Graphical abstract — (TIF) [file pone.0294062.s001.tif]
